# Mechanisms, Anti-Quorum-Sensing Actions, and Clinical Trials of Medicinal Plant Bioactive Compounds against Bacteria: A Comprehensive Review

**DOI:** 10.3390/molecules27051484

**Published:** 2022-02-22

**Authors:** Abdelhakim Bouyahya, Imane Chamkhi, Abdelaali Balahbib, Maksim Rebezov, Mohammad Ali Shariati, Polrat Wilairatana, Mohammad S. Mubarak, Taoufiq Benali, Nasreddine El Omari

**Affiliations:** 1 Laboratory of Human Pathologies Biology, Department of Biology, Faculty of Sciences, Genomic Center of Human Pathologies, Mohammed V University in Rabat, Rabat 10106, Morocco; 2 Centre GEOPAC, Laboratoire de Geobiodiversite et Patrimoine Naturel, Université Mohammed V de Rabat, Institut Scientifique de Rabat, Rabat 10106, Morocco; chamkhi.imane@gmail.com; 3 Agrobiosciences Program, University Mohammed VI Polytechnic, Lot 660, Hay Moulay Rachid, Ben Guerir 43150, Morocco; 4 Laboratory of Biodiversity, Ecology and Genome, Faculty of Sciences, Mohammed V University, Rabat 10106, Morocco; balahbib.abdo@gmail.com; 5 Department of Scientific Research, V. M. Gorbatov Federal Research Center for Food Systems, 26 Talalikhina St., 109316 Moscow, Russia; rebezov@yandex.ru; 6 Biophotonics Center, Prokhorov General Physics Institute of the Russian Academy of Science, 119991 Moscow, Russia; 7 Department of Scientific Research, K.G. Razumovsky Moscow State University of Technologies and Management (The First Cossack University), 109004 Moscow, Russia; shariatymohammadali@gmail.com; 8 Department of Clinical Tropical Medicine, Faculty of Tropical Medicine, Mahidol University, Bangkok 10400, Thailand; 9 Department of Chemistry, The University of Jordan, Amman 11942, Jordan; 10 Environment and Health Team, Polydisciplinary Faculty of Safi, Cadi Ayyad University, Safi 46030, Morocco; taoufiq.benali@uca.ac.ma; 11 Laboratory of Histology, Embryology and Cytogenetic, Faculty of Medicine and Pharmacy, Mohammed V. University in Rabat, B.P. 6203, Rabat 10000, Morocco; nasrelomari@gmail.com

**Keywords:** bacterial resistance to antibiotics, quorum sensing, bioactive compounds, clinical trial

## Abstract

Bacterial strains have developed an ability to resist antibiotics via numerous mechanisms. Recently, researchers conducted several studies to identify natural bioactive compounds, particularly secondary metabolites of medicinal plants, such as terpenoids, flavonoids, and phenolic acids, as antibacterial agents. These molecules exert several mechanisms of action at different structural, cellular, and molecular levels, which could make them candidates or lead compounds for developing natural antibiotics. Research findings revealed that these bioactive compounds can inhibit the synthesis of DNA and proteins, block oxidative respiration, increase membrane permeability, and decrease membrane integrity. Furthermore, recent investigations showed that some bacterial strains resist these different mechanisms of antibacterial agents. Researchers demonstrated that this resistance to antibiotics is linked to a microbial cell-to-cell communication system called quorum sensing (QS). Consequently, inhibition of QS or quorum quenching is a promising strategy to not only overcome the resistance problems but also to treat infections. In this respect, various bioactive molecules, including terpenoids, flavonoids, and phenolic acids, exhibit numerous anti-QS mechanisms via the inhibition of auto-inducer releases, sequestration of QS-mediated molecules, and deregulation of QS gene expression. However, clinical applications of these molecules have not been fully covered, which limits their use against infectious diseases. Accordingly, the aim of the present work was to discuss the role of the QS system in bacteria and its involvement in virulence and resistance to antibiotics. In addition, the present review summarizes the most recent and relevant literature pertaining to the anti-quorum sensing of secondary metabolites and its relationship to antibacterial activity.

## 1. Introduction

Infectious diseases are a group of pathologies caused by microorganisms, such as bacteria and viruses. Bacteria are considered the most implicated pathogens in infectious diseases. In fact, despite the discovery of antibiotics, bacteria have been able to develop resistance against these drugs via different mechanisms. Therefore, researchers have been searching for alternatives to conventional antibiotics. Within this context, natural substances, particularly those extracted from medicinal plants, constitute a source of drugs against various pathologies, including diabetes, cancer, inflammation, and pathologies linked to stress and microbial infections [1,2,3,4,5,6,7,8,9,10,11,12,13,14,15,16,17]. The antibacterial activity of these natural products is linked to different mechanisms of action, such as the increase in membrane permeability, the decrease in membrane integrity, and the disruption of efflux pumps [10,13,18,19]. However, the efficacy of these compounds is not significant for therapeutical applications, because some pathogenic strains continue to induce the problem of resistance, which, until recently, remained unrecognized. 

Importantly, it has been shown that bacteria–bacteria communications or quorum QS allow them to develop resistance against antimicrobials. In this regard, QS is an intercellular communication system between the same strain (self-communication) or different strains that co-ordinates the transcriptional regulation of genes responsible for several vital functions of these microorganisms [20,21,22]. Depending on the type and nature of the bacterial strain, the mode of regulation of this system involves certain signaling molecules [20,23,24,25]. 

Research findings indicated that Gram-positive bacteria use oligopeptides as auto-inducers to regulate gene expression. These molecules, after their secretion, bind to membrane receptors of the same bacteria, and the signal transduction can generate signaling pathways that result in the activation of transcription of a specific gene. On the other hand, Gram-negative bacteria regulate gene expression in the function of their density. They secrete other self-inducing molecules via the activation of the Lux operon, which regulates the transcription of main enzymes involved in QS mediators. These fluctuations of gene expressions occur according to the density and physiological state of bacteria [26].

Published research showed that QS regulates some major bacterial activities, including biofilm formation, pathogenicity, and antibiotic resistance. To limit the development of bacterial resistance to existing antibiotics and, subsequently, the emergence of infectious diseases, it is, therefore, necessary to search for anti-QS molecules [27,28]. For this, numerous studies were carried out in recent years [29,30,31]. Medicinal plants are rich sources of bioactive compounds, which target QS mediators with different modes of action [27,28], such as degradation, transcriptional inhibition of QS signal molecules, and the transport system between the intra- and extra-cellular media [32]. In this context, the effects of certain natural substances against QS are obtained from the phylogenetic relationships established between secondary metabolites of medicinal plants and QS intermediates [33,34,35]. Therefore, exploration of this path could assist in the discovery of anti-QS drugs. In this work, we have summarized the literature related to the role of the QS system in bacteria and its involvement in virulence and resistance to antibiotics, highlighting the effects of secondary metabolites, such as flavonoids, terpenoids, and phenolic acids, which exhibit anti-QS action. This will be beneficial in dealing with bacterial infections and with anti-resistant strains and assist in the development and discovery of novel drugs to treat these infections. 

## 2. Natural Products from Medicinal Plants as Antibacterial Drugs

In developing countries, infectious diseases are among the major causes of morbidity and mortality. In recent years, the scarcity of novel antimicrobials and resistance to currently available antibiotics has prompted pharmaceutical companies to develop new antibacterial drugs from natural substances. Genetic factors are behind the ability of numerous bacterial species to acquire resistance, which protects them from the antibacterial agents. This has been explained in several studies, which confirmed that the multi-resistance of some bacteria to drugs exists while being sensitive to other commonly used drugs [36,37]. The development of resistant bacterial species involves several factors, such as misuse of antibiotics in the treatment of bacterial infections, and also in animal feed as a growth promoter [38].

To supply the market with novel antibiotics, pharmaceutical companies have adopted strategies to increase the effectiveness of existing drugs or to restore their lost (or weakened) activity following bacterial resistance processes, which may be achieved by modifying their molecular composition [39]. In contrast, given the biodiversity of our planet, the search for antimicrobials of plant origin must have more interest from the point of the high number of medicinal plants used for therapeutic purposes by different populations all over the world for hundreds of years [39].

## 3. Classical Antibacterial Mechanisms of Natural Products Isolated from Medicinal Plants

### 3.1. Terpenoids

**Figure 1:**
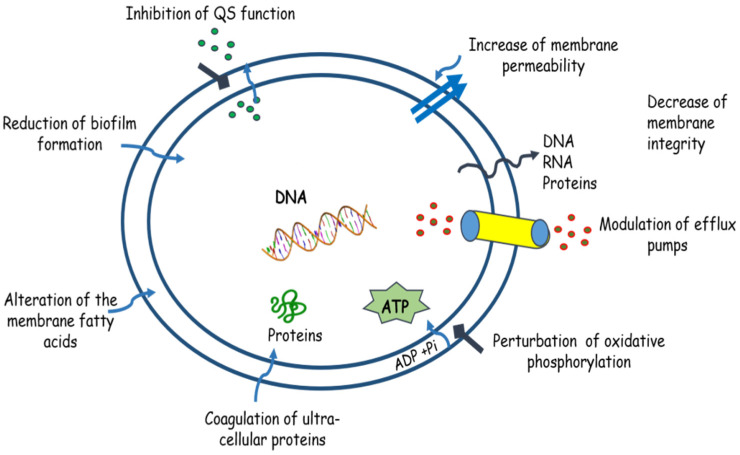
Antibacterial mechanisms of terpenoids. Terpenoids can exhibit their antibacterial actions via different mechanisms, such as the reduction in biofilm formation, alteration of the membrane fatty acids, coagulation of proteins, perturbation of oxidative phosphorylation, modulation of efflux pumps, the decrease in membrane integrity, the increase in membrane permeability, and inhibition of QS signaling.

The antibacterial mechanisms of terpenoids isolated from medicinal plants are multiple and include several related to the inhibition of bacterial growth, as depicted in Figure 1. 

**Figure 2:**
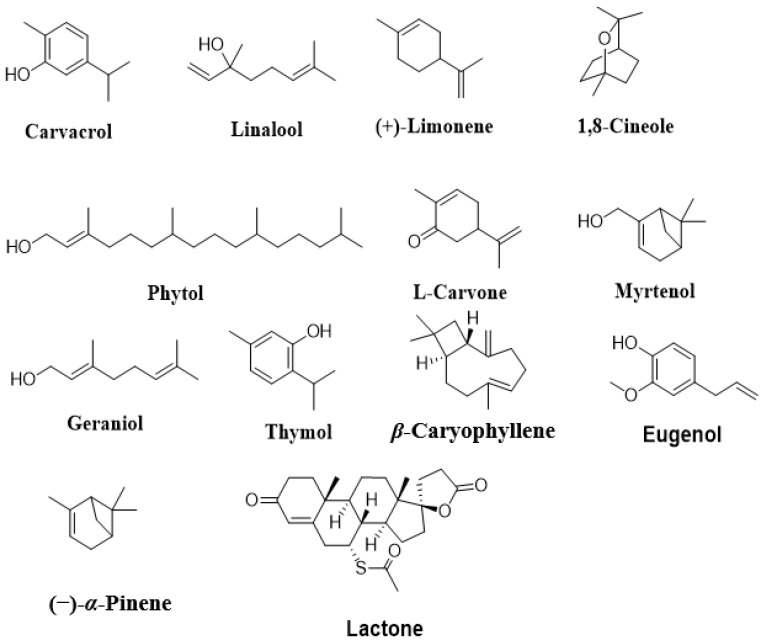
Chemical structures of terpenoids with antibacterial effects.

The main isolated terpenoids from medicinal plants, which exert antibacterial mechanisms, include l-carvone, phytol, limonene, linalool, β-caryophyllene, 1,8-cineole, myrtenol, geraniol, carvacrol, and thymol (Figure 2); these compounds exhibit promising antibacterial effects, as listed in Table 1.

Linalool, isolated from *Coriandrum sativum*, exhibits antibacterial activity against various strains, such as *Acinetobacter baumannii* [40], *Pseudomonas aeruginosa* [41], and *Salmonella* *Typhimurium* [42]. This compound exerts its effect against *A. baumannii* according to three mechanisms of action: QS, biofilm formation, and adhesion [40]. It exhibited antibiofilm activity via dispersion and inhibition of the formation of biofilms of the bacterium studied. In addition, this essential oil showed high antibacterial activity (MIC values between 2 and 8 μg/mL), with impaired bacterial adhesion and interference with the QS system. Against *P. aeruginosa*, linalool disrupted the respiratory chain and cell morphology, with bactericidal (MBC = 862 μg/mL) and bacteriostatic (MIC = 431 μg/mL) effects. It also exhibited the destructive power of membrane integrity, evidenced by the production of nucleic acids and a decrease in membrane potential [41]. To reduce the volatility of linalool and its low solubility/stability, Prakash and colleagues encapsulated linalool in nanoemulsions. These authors recorded a disruption of the membrane integrity in *S. Typhimurium*, with a decrease in biofilm formation (>11.5%) on the surface of pineapple sections [42].

Evaluation of the bactericidal activity of the main constituent of citrus EOs, (+)-limonene, against *E. coli* BJ4 (wild type) showed a decrease in bacterial resistance and cell wall permeability following sublethal thermal shock [43]. This was in line with the findings of Han et al. [43], who observed an increase in the conductivity and release of the intracellular contents of *L. monocytogenes*, indicating an alteration in the integrity of the cell wall. Additionally, the function of the respiratory complex can be inhibited by the disruption of energy and respiratory metabolism. This monoterpenoid also showed dose-dependent anticariogenic and antibiofilm activity against *S. mutans* and *S. pyogenes*, respectively [45], by preventing the formation and adhesion property of *S. pyogenes* biofilm, and inhibiting acid production and downregulating *vicR* gene expression in *S. mutans*. On the other hand, the pro-oxidant/antioxidant imbalance can be used as a therapeutic strategy against bacterial infections. In this context, the antibacterial activity of a chlorophyll component, phytol, was to induce a response to oxidative stress in *P. aeruginosa* [46]. Phytol produces excessive levels of intracellular reactive oxygen species (ROS), leading to a decrease in enzymatic antioxidants (glutathione peroxidase) and, consequently, inducing cell cycle arrest and severe DNA damage, ultimately leading to cell death.

The food industry is threatened by the emergence of pathogenic microorganisms. In this field, the QS system has been used by the bacterium *Hafnia alvei* to establish harmful virulence factors [58]. To deal with the threats of this opportunistic pathogen, Li et al. [47] treated it with l-carvone, a major compound of spearmint Eos, at sublethal concentrations. Results showed an inhibition of biofilm formation (52.41%) and the QS system, characterized by reduced synthesis of AHL (0.5 μL/mL). In contrast, an antibiofilm property of carvacrol has been demonstrated following its incorporation in a polyethylene-*co*-vinyl acetate film against *S. aureus* and *E. coli*. This led to disruption of the initial phase of bacterial attachment, which subsequently reduced the formation of biomass on the surfaces in comparison with the control (pure copolymer) [48].

Permeabilization and depolarization of the cytoplasmic membrane of *E. coli* growth was inhibited by carvacrol and its isomer (thymol) at a dose of 200 mg/L [50]. This agrees with the results of Churklam and colleagues, who found, in addition to these findings, an inhibition of the respiratory function of *Listeria monocytogenes* associated with degenerative changes [51]. This was also verified against *Salmonella Typhimurium* [49], with an MIC and MBC value of 312 µg/mL and inhibition of biofilm biomass (1.719 OD_550_) at 4 × MIC. On stainless steel and polypropylene, a decrease in bacteria counts was observed with carvacrol (4 × MIC) against the biofilm of this strain. With some monoterpenes, this reduction in biofilm biomass could reach 100%, as was the case with *S. aureus* treated with geraniol, which reduced bacterial viability at 1 mg/mL and biofilm formation at concentrations ranging from 0.5 to 4 mg/mL [52].

The antibacterial potential of certain terpenoids is little investigated, as is the case with myrtenol [53,54]. This bicyclic monoterpene alcohol exhibited promising results against methicillin-resistant *S. aureus* (MRSA) through biofilm inhibitory and anti-virulence activity against the main virulence factors (α-hemolysin, staphyloxanthin, autolysin, slime, and lipase) [53]. Likewise, myrtenol affected autolysis by releasing extracellular DNA, causing impairment of self-aggregation. Similarly, a bactericidal (MIC = MBC = 128 µg/mL) and antibiofilm action against *S. aureus* was noted with this molecule by blocking the synthesis of the cell envelope. The combination of myrtenol with conventional antibacterial agents highlighted these effects [54]. Another monoterpene constituent known as eucalyptol or 1,8-cineole, when tested against *Salmonella* sp. D194-2, altered the bacterial wall structure and downregulated the membrane protein genes at the mRNA level [55]. Finally, β-caryophyllene, in turn, exhibited significant anti-*S. mutans* effect [56]. Indeed, this bicyclic sesquiterpene inhibits cell growth and biofilm formation, with a decrease in the expression of *gtf* genes. Impairment of membrane permeability and integrity of *Bacillus cereus* was also induced by this molecule, subsequently leading to leakage of intracellular contents, causing cell death [57].

Using in vitro and in vivo experiments, Wan et al. [59] evaluated the antibacterial effect of patchouli alcohol against bacteria and drug-resistant bacterial strains. Results revealed that patchouli alcohol exhibits antibacterial activities against all bacteria tested. In this regard, both Gram-negative (25–768 μg/mL) and Gram-positive bacteria (1.5–200 μg/mL) were sensitive to this compound. Importantly, patchouli alcohol was active against certain drug-resistant bacteria, such as methicillin-resistant *Staphylococcus aureus* (MRSA). Results also demonstrated that patchouli alcohol at 100 and 200 mg/kg, could protect infected mice with MRSA, while, at a low dose of 50 μg/mL, could protect 80% of mice injected with MRSA. Furthermore, patchouli alcohol, isolated from *Pogostemonis Herba*, exhibited a selective antibacterial effect against *Helicobacter pylori* and was not active against the main normal gastrointestinal bacteria [60]. This antibacterial effect was superior to amoxicillin and associated with urease inhibitory potential.

Similarly, the potential anti-*Staphylococcus aureus* effect of andrographolide was demonstrated by Banerjee and colleagues [61]. This compound caused specific inhibition of intracellular DNA biosynthesis in a dose-dependent manner and mediated inhibition of biofilm formation by *S. aureus*. On the other hand, Wolska and collaborators [62] showed that oleanolic acid is active against some bacterial species, particularly *mycobacteria*. The study of the mechanism of its antibacterial activity showed that this acid affects bacterial gene expression, inhibits the formation and maintenance of biofilms, and causes cell autolysis and peptidoglycan turnover. The same acid displayed antibacterial activities against the tested bacteria, thus 1/4 MIC can reduce bacterial biofilm formation [63].

### 3.2. Antibacterial Actions of Flavonoids

The likelihood of bacterial strains becoming resistant increases steadily as they coexist with substitute compounds for the majority of existing antibacterial agents. It is, therefore, obvious to move towards other synthetic compounds devoid of these substitutions, and to find new natural substances or new molecular targets. In this regard, flavonoids have protected plants as well as humans against pathogens through their antibacterial potential [64]. Most pathogens cannot develop resistance to natural substances, which make them a therapeutic alternative in bacterial infections [65]. In this sense, we have focused on the different antibacterial mechanisms and therapeutic targets of various flavonoids.

#### 3.2.1. Inhibition of Cell Envelope (Wall) Synthesis

The FAS-II pathway is essential in the biogenesis of the envelope in Gram-negative bacteria, which makes it a prime therapeutic target for numerous antibacterial agents [66]. Inhibition of this pathway has in fact blocked the generation of signaling molecules, such as AHLs, implicated in cellular communication [67]. In another investigation on *Helicobacter pylori*, three flavonoids: apigenin, sakuranetin, and quercetin, inhibited β-hydroxyacyl-ACP dehydrase, one of the FAS-II constituents, with IC_50_ values of 11.0 ± 2.5, 2.0 ± 0.1, and 39.3 ± 2.7 μM, respectively [68]. On the other hand, epigallocatechin gallate (EGCG) and DL-cycloserine can synergistically inhibit synthesis of cell wall (MIC = 128 μg/mL), while catechins can bind to the peptidoglycan layer of this cell wall [69]. Mycobacteria contain fatty acids called mycolic acids in their cell wall, synthesized by the mammalian type FAS-I and the bacterial type FAS-II, allowing them to have a high resistance. Findings by Li et al. [70] showed that two flavone derivatives: luteolin (IC_50_ = 2.52 ± 1.0 μM) and baicalein (IC_50_ = 111.69 ± 2.29 μM), exhibit inhibitory activity against FAS-I by targeting this pathway. Other flavonoids, such as taxifolin (IC_50_ = 41.16 ± 0.59 μM), hesperetin (IC_50_ = 68.86 ± 4.49 μM), fisetin (IC_50_ = 18.78 ± 0.49 μM), myricetin (IC_50_ = 27.18 ± 0.24 μM), morin (IC_50_ = 2.33 ± 0.9 μM), quercetin (IC_50_ = 4.29 ± 2.5 μM), and kaempferol (IC_50_ = 10.38 ± 0.07 μM), exhibited similar antibacterial activities using the same mechanism of action [70].

#### 3.2.2. Inhibition of Nucleic Acid Synthesis

In addition to the aforementioned therapeutic strategies, inhibition of DNA topoisomerases can also constitute an important therapeutic target against bacteria. At a dose of 0.1 µM, genistein altered the cell division of *Vibrio harveyi*, which subsequently inhibited its growth [71]. Similarly, DNA gyrase, a protein involved in the replication of the bacterial circular chromosome, was inhibited (IC_50_ = 3.3 μg/mL) by ellagic acid in a treatment against *E. coli*. Other flavonoids, such as apigenin, 3,6,7,3′,4′-pentahydroxyflavone, and quercetin, exhibited marked inhibitory activities against DNA gyrase, with IC_50_ values of 67.6, 55, and 47 μg/mL, respectively [72]. This last flavonoid also blocked *E. coli* DNA supercoiling (*K*_D_ = 15 μM) [73]. Additionally, EGCG inhibited the growth of three bacterial strains: *Mycobacterium tuberculosis*, *E. coli*, and *Streptomonas* *maltophilia*, by inhibiting the activity of an enzyme responsible for DNA synthesis called dihydrofolate reductase (DHFR) [74,75].

#### 3.2.3. Inhibition of Bacterial Motility

In order to multiply, bacteria need to colonize and invade host tissues by displacement and adhesion through their surface components and biofilms [76]. Flavonoids are proposed to prevent this multiplication (adhesion and colonization) by paralyzing bacteria via blocking their motility [77]. Numerous flavonoids, including luteolin (IC_50_ = 12.5–50 μg/mL), naringenin (IC_50_ = 100 μg/L), quercetin (IC_50_ = 0.085 μg/mL), EGCG (IC_50_ = 40 μg/mL), nobiletin (IC_50_ = 100 μM), sinensetin (IC_50_ = 100 μM), and morin (IC_50_ = 30 μg/mL), inhibited bacterial motility (twitching motility) [78]. Moreover, other flavonoids have shown antibiofilm and anti-QS effects on *P. aeruginosa* [79]. Interestingly, quercetin was the constituent that strongly inhibited its twitching motility (MIC = 0.085 μg/mL) as well as biofilm formation (95%).

#### 3.2.4. Inhibition of Biofilm Formation

As previously mentioned, bacteria form biofilms on surfaces to adhere, grow, and subsequently synthesize polymers that can induce alterations related to gene transcription and growth rate [80]. In this respect, it is difficult to target bacteria within a fully formed biofilm, as they receive the elements necessary for their survival through water channels that irrigate the biofilm [80]. Phloretin acted as an antibiofilm agent against *E. coli* [81], indicating the inhibitory potential of hydrophilic flavonoids against biofilm formation [82]. This agrees with the findings of Vikram and colleagues, who recorded a decrease in the size of biofilms formed by *V. harveyi* and *E. coli* via the action of sinensetin, quercetin, kaempferol, rutin, naringin, neohesperidin, naringenin, neoeriocitrin, and apigenin [83]. EGCG also destroyed the established biofilm of *Porphyromonas gingivalis* [84] and *E. faecalis* [85].

### 3.3. Antibacterial Actions of Phenolic Acids

Phenolic acids are a group of secondary metabolites that are widely found in medicinal plants. These compounds exhibit remarkable antibacterial properties with different mechanisms, including the perturbation of influx of protons, decreasing cell viability, and increasing cell membrane permeability. Through their properties of absorption, digestion, and metabolism in the circulatory system, the bioavailability of phenolic acids is linked to the intensity of their pharmacological effects. These compounds represent the main polyphenols and are the source of multiple biomolecules used in cosmetics, food, and therapeutic industries [86]. In microorganisms, increased cell membrane permeability is generally an important mechanism of action for a wide range of antimicrobials. This mechanism of action was adopted by Campos et al. [87] to assess the activity of numerous phenolic acids against Oenococcus oeni and Lactobacillus hilgardii, two wine lactic acid bacteria, by measuring the influx of protons, cell viability, and the efflux of phosphate and potassium. Therefore, hydroxycinnamic acids induced more ion loss and proton influx than hydroxybenzoic acids. A decrease in cell viability in both strains tested was noted after exposure to phenolic acids [87].

In 2015, Oh and Jeon [88] investigated the inhibitory potential of 12 phenolic acids (salicylic acid, gallic acid, benzoic acid, *p*-hidroxybenzoic acid, tannic acid, protocatechuic acid, syringic acid, *p*-coumaric acid, sinapic acid, ferulic acid, cinnamic acid, and vanillic acid) in association with two synthetic antibiotics (ciprofloxacin or erythromycin) against *Campylobacter jejuni* of poultry or human origin. Results showed that certain combinations have synergistic inhibitory effects. Authors of this study attributed the modulatory effects of certain acids (*p*-coumaric acid and gallic acid) on antibiotic resistance to reduction in membrane transporter transcription and to disturbances in membrane envelope permeability. Finally, in *C. jejuni*, gallic acid exhibited high transcription reductions in *CmeABC*, acting as a multi-drug efflux system responsible for the resistance of this bacterium. A year later, other researchers examined the activity of tannic acid alone or in combination with norfloxacin, a broad-spectrum antibiotic, against the *S. aureus* strain [89]. This phenolic acid inhibited overexpression of the *norA* gene encoding the efflux transporter protein *NorA*, with an MIC value of 0.512 mg/mL. Tannic acid combined with norfloxacin also inhibited the growth of the tested strain synergistically. Likewise, this acid inhibited the *NorA* efflux pump, indicating a modulation of antibiotic resistance [89].

Research findings [90] indicated that caffeic acid alone exhibits antibacterial activity, with MIC values ranging from 256 to 1024 µg/mL, against a reference strain of *S. aureus*, while it potentiated the antibacterial effect of clindamycin, cefoxitin, vancomycin, and erythromycin. Other phenolic acids (*p*-coumaric, ellagic, protocatechuic, gallic acid, vanillic, and syringic), isolated from grape pomace extracts, inhibited the growth of *E. coli* and *S. aureus*, with MIC values ranging from 0.2 to 2.5 and 0.062 to 3 mg/mL, respectively [91]. *Salmonella Enteritidis* and *Listeria monocytogenes* were also inhibited by ellagic acid (0.2 mg/mL) identified in *Vaccinium corymbosum* L. [92]. In contrast, two studies investigating the activity of gallic acid against *Helicobacter pylori* [93] and *E. coli* [94] were conducted by Díaz-Gómez and his colleagues. These researchers showed that the growth of both bacteria was strongly inhibited by this molecule at doses of 0.2 and 3.25 mg/mL, respectively [94].

Other molecules, such as gallic acid, vanillic acid, caffeic acid, protocatechuic acid, and *p*-coumaric acid, were effective against methicillin-resistant *S. aureus* (MRSA), with MIC values of 2.05, 2.05, 2.05, 4.09, and 1.30 mg gallic acid equivalent/mL (GAE/mL), respectively [95]. Thus, phenolic acids can be suggested as food preservation due to the chemical structure of these acids [96]. In fact, the antibacterial activity has been improved following the increase in the length of the alkyl chain [97]. Additionally, two phenolic acids: hydroxybenzoic and hydroxycinnamic acids, exhibited an antibacterial activity dependent on the number of hydroxyl (−OH) and methoxy (−OCH_3_) functional groups [98]. In addition, Bouarab-Chibane et al. [99] demonstrated that ferulic and gallic acids cause disruption of membrane integrity and leakage of intracellular elements. Moreover, other studies attributed the antibacterial activity of coumarins to their capacity to induce reduction in the rate of cellular respiration and inhibition of the bacterial division protein FtsZ [99,100].

Certainly, the structural variability between the different polyphenols has an impact on their antibacterial potential. Data collected and discussed in this review indicate that phenolic acids are characterized by a strong antibacterial activity compared to flavonoids having a large molecular structure. The strong interaction of phenolic acids with the active sites of bacteria was related to their reduced molecular size [91,95,101]. Furthermore, research findings showed that phenolic acids induce cell death by inhibiting bacterial growth via acidification of the cytoplasm [87]. This is inversely proportional to the pH values [102]. This is due to the fact that pH exerts a load on ring substitutions (−OH and −OCH_3_), the −COOH group, and side-chain saturation. In addition, the antibacterial effect decreases with the decrease in double bonds in hydroxycinnamic acids [87]. To improve the antibacterial activity of terpenoids, flavonoids, and phenolic acids, it is necessary to study, in vitro and in vivo, the possible synergetic antibacterial effects of formulation/combination of these molecules between themselves and between clinically prescribed antibiotics. In addition, characterization of the underlying mechanisms of action of these molecules is an interesting approach to improve their efficiency; for example, nano-encapsulate of certain molecules can facilitate their penetration of the bacterial wall.

## 4. Secondary Metabolites of Medicinal Plants as Anti-Quorum-Sensing Agents

### 4.1. Quorum-Sensing Systems in Bacteria

When a bacterial community reaches a high level, signaling molecules will be synthesized subsequently, and this is called QS. For the expression of these molecules to take place, the cell density must be high. Therefore, a set of genes are then activated by these QS molecules for the biosynthesis of proteins involved in pathogenicity and antibiotic resistance [103,104]. The expression of QS molecules, from a biochemical point of view, differs depending on the cells (Gram− or Gram+) [105]. Regarding Gram-positive bacteria, the main function of the QS system is to ensure the synthesis of intracellular molecules called self-inducing peptides (AIP), which will be transported outside the cell in the form of oligopeptides capable of binding to external membrane receptors rich in histidine. Specific activation of gene expression is provided by signaling pathways activated by signal transduction; a given signal peptide specifically regulates the transcription of a gene [105]. However, with Gram-negative bacteria, self-inducing molecules are secreted from a parent molecule called *N*-acyl homoserine lactones (AHL). These bacteria, at high cell density, activate the transcription of the Lux operon encoding the transcription of enzymes of the signal synthase (LuxI) family responsible for the synthesis of AHL. Depending on the bacterial density, these molecules can rejoin the intracellular medium to regulate the expression of genes, in a manner dependent on the extracellular medium [105]. In fact, in order to selectively activate the transcription of the target genes, the AHL molecule diffuses into the intracellular medium and interacts with the regulators of transcription.

### 4.2. Action of Secondary Metabolites on QS

**Figure 3:**
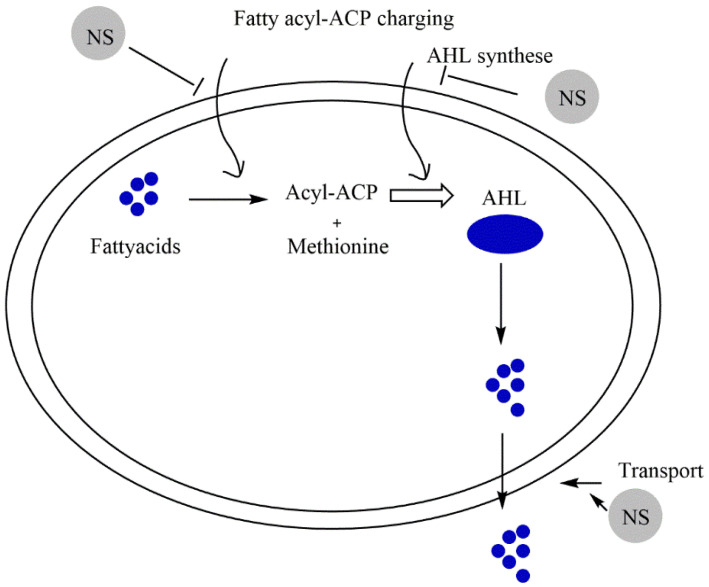
Natural substances targeted QS in bacteria via inhibition of AHL signal generation. Natural bioactive compounds can induce the inhibition of the synthesis of the substrate for the AHL synthase (fatty acyl-acyl carrier protein: acyl-ACPs), the inhibition synthesis of AHL, and the inhibition of the AHL transport. Abbreviations: AHL, *N*-acyl homoserine lactone.

**Figure 4:**
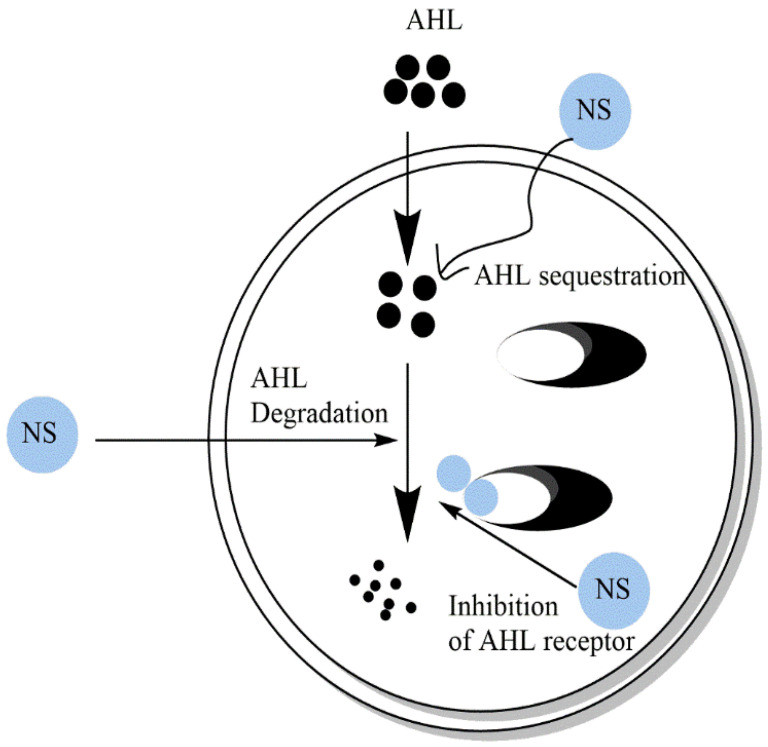
Natural substances targeted quorum sensing in bacteria via inhibition of the signal reception. They can induce AHL degradation, AHL sequestration, and competition on AHL receptor AHL-mimetic compounds.

Recent investigations showed that several natural products exhibit important effects against QS mediators. In this review, the focus will be on the anti-QS action of secondary metabolites secreted from medicinal plants. These compounds mainly belong to terpenoids, phenolic acids, and flavonoids. The general mechanisms of these natural substances include inhibition of the generation of QS mediators (Figure 3) and QS mediators’ reception (Figure 4). 

#### 4.2.1. Terpenoids

As described in other parts of this review, terpenoids or EOs exhibit remarkable antibacterial effects via different mechanisms, including inhibition of QS. Indeed, as listed in Table 2, numerous terpenoids, such as carvacrol, linalool, d-limonene, and α-pinene, display inhibitory activities through different mediators of QS.

Along this line, eugenol exhibits important effects against biofilms of *Pseudomonas aeruginosa*, *Proteus mirabilis*, and *Serratia marcescens* clinical isolates [112], and against methicillin-resistant *Staphylococcus aureus* isolated from food handlers [114]. Remarkably, other published work revealed that eugenol inhibited the production of virulence factors, such as violacein, elastase, pyocyanin, and biofilm formation, in *Pseudomonas aeruginosa* [112,113,114,115,116]. It additionally inhibited QS-controlled gene expression in *Pseudomonas aeruginosa* QSIS-lasI and Chromobacterium violaceum CV026 biosensors [113]. On the other hand, eugenol caused an important reduction in biofilm formation on PAO1 (65.6%) and a remarkable effect against QS signals (AIs) (*p* < 0.001) [115]. Recently, other investigations [116] showed that eugenol reduces 50% of violacein production in *Chromobacterium violaceum* at sub-MIC of 0.2 mg/mL, as well as the production of *N*-(3-oxododecanoyl)-l-homoserine lactone (3-oxo-C12-HSL) and C4-HSL *N*-acyl homoserine lactone signal molecules, pyocyanin, and swarming motility in *P. aeruginosa.* Moreover, eugenol inhibited the expression of QS synthase genes with an expression level of 65% and 61% for *lasI* and *rhlI*, respectively, and 65% for *rhlA* gene, as well as the biofilm formation (36%) [116].

In a similar fashion, carvacrol (2-methyl-5-(1-methylethyl)-phenol) showed efficacy against biofilm growth and QS. Indeed, recent studies have shown that carvacrol [107] inhibits the formation of biofilms in *Pseudomonas aeruginosa* at very low concentrations (0.9–7.9 mM) and, at the same time, reduces synthesis of pyocyanin and violacein at the these concentrations, with a percentage of 60 and 50% at the concentration of 3.9 mM and 0.7 mM, respectively [107]. More recently, another study showed that carvacrol reduces the virulence of *Pseudomonas aeruginosa* via inhibition of LasI expression and concomitant reduction in lasR expression, biofilm formation, and swarm motility [108]. In this context, the inhibitory action of biofilm formation has already been demonstrated in *Chromobacterium violaceum* ATCC 12472, *Salmonella enterica* subsp, *Typhimurium* DT104, and *Staphylococcus aureus* 0074 at sublethal concentrations (<0.5 mM) by reducing the expression of cviI, violacein, and chitinase [106].

Diterpenes, such as phytol, showed the ability to inhibit biofilm growth and QS in *Pseudomonas aeruginosa* PAO1 and *Serratia marcescens* [86,87,88]. At a concentration of 10 μg/mL, phytol inhibited production of prodigiosin (92%), QS-mediated protease (68%), and biofilm formation (64%) of *Serratia marcescens* [118]. Using the same bacterial strain and concentration, results revealed that phytol lowers the level of biofilm formation, lipase, and hemolysin production, and inhibits the swarming motility and EPS productions. This compound also downregulated the *fimA*, *fimC*, *flhC*, *flhD*, *bsmB*, *pigP*, and *shlA* gene expressions, and reduced the level of virulence enzymes (lipase and protease productions) [119]. In another study, results showed that phytol reduces the formation of *Pseudomonas aeruginosa* biofilm in the range of 74.00–84.33%. It also effectively reduced *P. aeruginosa* twitching and flagella motility, and inhibited the pyocyanin production (51.94%) [117].

Phytol is a diterpene, which has demonstrated anti-QS activity. In this regard, this compound inhibited the growth of the biofilm and the detection of quorum in *Pseudomonas aeruginosa* PAO1 and *Serratia marcescens* [117,118,119]. At a concentration of 10 µg/mL, phytol inhibited the production of prodigiosin (92%) and QS-mediated protease (68%), and biofilm formation (64%) in *Serratia marcescens* [118]. In the same bacterial strain, phytol decreased the level of biofilm formation, lipase and hemolysin production, and also inhibited swarm motility. These effects are associated with the regulation of the expression of certain genes (such as the *fimA*, *fimC*, *flhC*, *flhD*, *bsmB*, *pigP*, and *shlA* genes) and reduction in the activity of virulence enzymes (lipase and protease) [119]. Moreover, using *Pseudomonas aeruginosa* as a study model, researchers showed that phytol reduces biofilm formation, diminishes flagella motility, and inhibits pyocyanin production [117].

Sesquiterpene lactone is another terpene, which has also shown anti-QS activity. In fact, this molecule exerted an inhibitory activity against QS mediators in *Pseudomonas aeruginosa* ATCC 27,853 and *Chromobacterium violaceum* [109,110,111]. In this respect, sesquiterpene lactone [111] inhibited QS (QSI ≥ 80%) at 1.31 mg/mL in *Pseudomonas aeruginosa*. Research findings showed that the action of six sesquiterpene lactones belonging to the chemical families of goyazensolide and isogoyazensolide inhibit the production of AHL at the concentration of 100 µg/mL. These results indicated that sesquiterpene lactones are good candidates for the development of new antimicrobial agents. Similarly, oleanolic aldehyde coumarate exhibited inhibitory activities against *P. aeruginosa* biofilms via inhibition of las, rhl, and AHL expression, as well as by reduction in *lasI/R*, *rhlI/R* expression, and gacA [122]. Other terpenoids, such as linalool, inhibited the biofilm formation of *A. baumannii* and modified the adhesion of this strain to surfaces. This phenotype is linked to the interference of linalool with the QS system [40,120]. In the meantime, using *E. coli* as a study model, *D*-limonene nanoemulsion inhibited biofilm formation by suppressing the production of extracellular polymeric substances (EPS) and decreasing the capacity swarming. On the other hand, (−)-α-pinene (at a concentration of 250 mg/L) has recently shown a significant reduction in QS *Campylobacter jejuni* signaling of >80% [121].

#### 4.2.2. Flavonoids 

**Figure 5:**
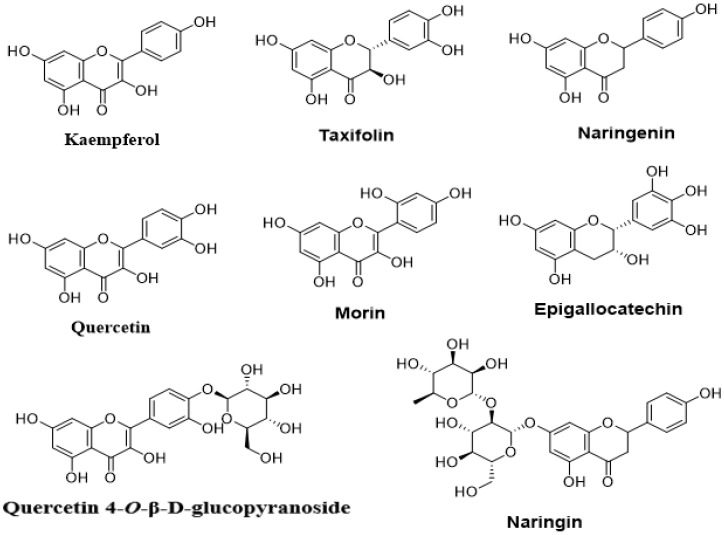
Chemical structures of flavonoids with anti-quorum-sensing effects.

Flavonoids constitute the second group of medicinal plant secondary metabolites. Some investigations that have been carried out recently showed that this chemical group exhibits an antibacterial effect via several actions, including inhibition of QS and its major phenotypes, such as the formation of biofilm. Listed in Table 3 are investigations showing the effects of flavonoids (Figure 5) against QS and biofilm formation. 

Epigallocatechin showed antibiofilm activity against *Salmonella typhimurium*, with downregulation of the *di A* and *luxS* genes [127,128]. Research findings showed that epigallocatechin decreases the production of Streptococcus mutans biofilms at a concentration of 250 μg/mL. Furthermore, epigallocatechin disrupted the QS activity, reduced motility and biofilm formation, and decreased AI-2 activity [126]. In addition, epigallocatechin has also shown inhibitory activities of QS and biofilm formation against *Burkholderia cepacia* and *Staphylococcus aureus* [123], *Listeria monocytogenes* [125], and *Eikenella corrodens* [124]. On the other hand, at concentrations of 100 and 200 µg/mL, naringenin inhibited the growth and biofilm formation of *S. mutans*, increased the surface hydrophobicity of *S. mutans*, reduced bacterial aggregation, and regulated downward mRNA expression of gtfB, gtfC, comD, comE, and luxS [130]. Furthermore, results indicated that this compound inhibits swimming and swarming motility in Chromobacterium violaceum and is associated with inducing transcription levels of yenR, flhDC, and fliA [129].

Quercetin has been investigated by several researchers for its anti-QS activities [79,83,131,132,133,134,135,136]. Results revealed that the actions of quercetin against QS are diverse and multiple, and depend on the bacterial strain tested and the experimental method used. Within this context, quercetin exerts antagonistic effects on bacterial signaling. Moreover, it suppresses biofilm formation, as has been demonstrated in *Escherichia coli* O157: H7 and *Vibrio harveyi* [83]. In addition, quercetin inhibited the QS-controlled virulence factors, such as violacein, elastase, and pyocyanin in *Chromobacterium violaceum* CV12472 and *Pseudomonas aeruginosa* PAO1 [133]. Using biofilm formation assay, Ouyang and colleagues [136] reported that quercetin decreases adhesion and biofilm formation in *Pseudomonas aeruginosa*, as well as swarming motility and expression of biofilm-associated genes. Quercetin also showed significant reduction in QS-dependent phenotypes, including violacein production, biofilm formation, and exopolysaccharide (EPS) production in *Chromobacterium violaceum* CV026, as well as motility and alginate production in a concentration-dependent manner [131]. Ouyang et al. [132] reported that quercetin exhibits antibiofilm activities against *Pseudomonas aeruginosa* strain PAO1, as well as inhibition of production of virulence factors, including pyocyanin, protease, and elastase at low concentrations. Furthermore, the expression levels of *lasI, lasR, rhlI*, and *rhlR* were reduced by quercetin at a concentration of 16 μg/mL. This compound also inhibited the QS circuitry by interacting with transcriptional regulator LasR in *Pseudomonas aeruginosa* [134]. 

Other flavonoids have also shown important anti-QS activities. In this respect, Hernando et al. [140] indicated that naringenin inhibits the expression of QS-regulated genes, as well as the production of the QS-regulated virulence factors, pyocyanin and elastase, in *Pseudomonas aeruginosa* strains. In a similar fashion, morin exhibited significant biofilm inhibition, reduced motility and spreading, and EPS production of *Staphylococcus aureus* [139]. Meanwhile, kaempferol inhibited biofilm formation by 80% at a concentration of 64 μg/mL and reduced the activity of *Staphylococcus aureus* sortase A (SrtA) and the expression of adhesion-related genes [138]. On the other hand, taxifolin exerted a significant decrease in the production of pyocyanin and elastase in *P. aeruginosa* without affecting bacterial growth. This compound also reduced the expression of several QS-controlled genes (i.e., *lasI*, *lasR*, *rhlI*, *rhlR*, *lasA*, *lasB*, *phzA1*, and *rhlA*) in *P. aeruginosa PAO1* [137].

#### 4.2.3. Phenolic Acids 

**Figure 6:**
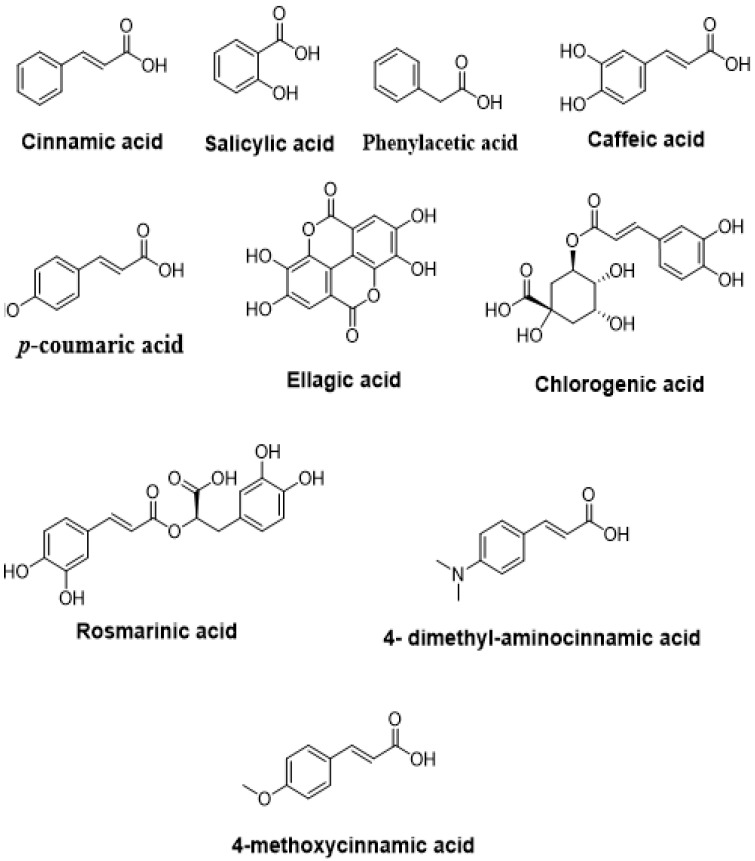
Chemical structures of phenolic acids with anti-quorum-sensing effects.

Phenolic acids are also secondary metabolites secreted by several natural resources, including medicinal plants. Several investigations showed that these phenolic compounds (Figure 6) exhibit remarkable anti-QS effects. Shown in Table 4 are the anti-QS effects of phenolic acids.

Salicylic acid was reported to interfere with the QS system of two *Pectobacterium* species, *P. aroidearum* and *P. carotovorum* ssp. *Brasiliense*, and affected QS machinery, consequently altering the expression of bacterial virulence factors [149]. It also inhibited the expression of QS genes, including *expI*, *expR*, *PC1_1442* (*luxR* transcriptional regulator), and *luxS* (a component of the AI-2 system), and reduced the level of the AHL signal. Using motility and AHL production tests, treatment with salicylic acid significantly reduced the biofilm formation by decreasing twitching and swarming motility and AHL production in *Pseudomonas aeruginosa* [147]. This activity was also confirmed by other researchers [148]. In another study, salicylic acid reduced the AHL production and biofilm formation in *Agrobacterium tumefaciens by* modulation of 103 gene families involved in virulence [146].

Similarly, rosmarinic acid (RA) at 750 μg/mL inhibited biofilm formation and reduced the QS-mediated hemolysin, lipase, and elastase production in *A. hydrophila* strains. It additionally downregulated the virulence genes, such as *ahh1*, *aerA*, *lip*, and *ahyB* [143]. Using molecular docking, researchers Corral-Lugo et al. [142] showed that RA bound to the QS regulator RhlR of the *Pseudomonas aeruginosa* PAO1 and competes with the bacterial ligand N-butanoyl-homoserine lactone (C4-HSL), and stimulated a greater increase in RhlR-mediated transcription than that of C4-HSL. In *P. aeruginosa*, RA induced the QS-dependent gene expression and increased biofilm formation and the production of the virulence factors pyocyanin and elastase. In another study, results revealed that RA induces the expression of 128 genes, including numerous virulence factor genes, and triggered a broad QS response in *Pseudomonas aeruginosa* PAO1. It also induced seven sRNAs that were all encoded in regions close to QS-induced genes [144]. Using the same model organism, researchers confirmed this activity [141].

Cinnamic acid is another type of phenolic acid that has documented biofilm and QS inhibitory activities. At sublethal concentration, cinnamic acid effectively inhibited both the production of the QS-dependent virulence factors and biofilm formation in *P. aeruginosa* without affecting the viability of the bacterium [150]. In addition, findings showed that cinnamic acid affects the QS machinery of the two species (*Pectobacterium aroidearum* and *Pectobacterium carotovorum* ssp. *brasiliense*), consequently altering the expression of bacterial virulence factors [149]. Furthermore, cinnamic acid inhibited the expression of QS genes, including *expI*, *expR*, *PC1_1442* (*luxR* transcriptional regulator), and *luxS* (a component of the AI-2 system), and reduced the level of the AHL signal. In a similar fashion, two cinnamic acid derivatives, 4-dimethylaminocinnamic acid (DCA) and 4-methoxycinnamic acid (MCA), exhibited anti-QS and antibiofilm activities against *Chromobacterium violaceum* ATCC12472 [151]. Additionally, both DCA (100 μg/mL) and MCA (200 μg/mL) inhibited the levels of N-decanoyl-homoserine lactone (C10-HSL) and reduced the production of certain virulence factors in *C. violaceum*, including violacein, hemolysin, and chitinase. Moreover, DCA and MCA downregulated the QS-related metabolites, such as ethanolamine and L-methionine, suppressed the expression of two QS-related genes (*cviI* and *cviR*), and inhibited the biofilm formation.

For chlorogenic acid, researchers found that CA inhibits the formation of biofilm in *Pseudomonas aeruginosa*, the ability of swarming, and virulence factors, including protease and elastase activities, and rhamnolipid and pyocyanin production [145]. Chlorogenic acid also exhibited similar inhibitory effects in *Chromobacterium violaceum* on its biofilm formation, swarming motility, chitinolytic activity, and violacein production. Similarly, *p*-coumaric acid inhibited QS responses of *Agrobacterium tumefaciens* NTL4, *Chromobacterium violaceum* 5999, and *Pseudomonas chlororaphis* with no effect on cell viability [152]. Using a qualitative QS inhibition assay, researchers showed that, at 0.2 mg/mL, chlorogenic acid suppresses the QS in *Chromobacterium violaceum* (CECT 494) by inhibiting the violacein [153]. Caffeic acid was reported to have anti-QS and antibiofilm effects in *Staphylococcus aureus* by inhibiting the production of α-hemolysin by this microorganism [154]. In terms of biofilm formation, ellagic acid and phenylacetic acid were shown to be effective against *Burkholderia cepacia* [123] and *Pseudomonas aeruginosa* [155].

## 5. Clinical Investigations of Natural Compounds Isolated from Medicinal Plants

### 5.1. Clinical Investigations of Terpenoids

In order to validate the tolerance, efficacy, and safety of a treatment, clinical trials are carried out in human medical therapy after preclinical studies (Table 5). Numerous clinical studies have been performed with the aim of discovering new natural constituents with antibacterial properties. However, these properties are little explored with terpenoids at the clinical level [156,157]. In 2011, the impact of a vaginal douching based on two monoterpenes (thymol + eugenol) against bacterial vaginosis (BV) was studied in a randomized, multicenter parallel group trial in 221 women [158]. At the rate of showering/day for a whole week, positive results have been observed, namely, a decrease in inflammatory signs, vaginal pH, and intensity of itching. Certain types of vaginal inflammation, such as BV, may be caused by the natural overgrowth of vaginal bacteria, while combination therapy between the two monoterpenes of this study may be advised for the management of minor vaginal infections. Indeed, the combination of treatments often led to remarkable results [158]. 

To highlight the combined effect of thymol with chlorhexidine, a broad-spectrum antiseptic, against two bacteria of the oral cavity (*S. mutans* and lactobacilli), a study was carried out including 90 disabled children, randomly divided into three groups [159]. After one month and six months of treatment, a reduction in bacterial growth compared to the control group was noted, suggesting that this combination may be recommended for improving oral hygiene and preventing dental caries in children with disabilities. In a randomized, double-blind, and placebo-controlled trial, 33 patients with *Helicobacter pylori* infection were treated with β-caryophyllene (126 mg/day) for 8 weeks to determine eradication rates and inflammation levels [157]. Results showed relief of epigastralgia, reduced severity of nausea, and decreased levels of proinflammatory cytokines (IL-1β), suggesting that this chemical class can be used in medicinal preparations for the treatment of different bacterial infections in several sectors, such as cosmetics and food industries.

### 5.2. Clinical Investigations of Flavonoids

Urinary tract infection is a disease that affects the kidneys and/or bladder and is often bacterial in origin, particularly related to *E. coli*. Consumption of cranberries (*Vaccinium macrocarpon* Ait.) in traditional medicine represents an alternative in the prevention of this type of infection. This use is justified by the high contents of proanthocyanidins (PACs), having the capacity to inhibit adhesion of *E. coli* to the epithelial cells of the bladder. In some clinical trials, these molecules were among the first flavonoids investigated against bacterial infections [160,161]. 

In the first trial, 32 adult volunteers of different nationalities (Spanish, French, Japanese, and Hungarian) received a diet rich in PACs (72 mg/day) in order to evaluate (ex-vivo) their urinary bacterial antiadhesion effect in a randomized, double-blind, placebo-controlled study, as well as to test the impact of this regimen on *E. coli* virulence using an in vivo model of *Caenorhabditis elegans* [160]. Results showed a dose-dependent inhibition of bacterial adhesion, with a weakening of the activity of *E. coli* to kill *C. elegans* being obtained after treatment with the cranberry powder diet. This confirms the benefits of PACs in preventing *E. coli* virulence and its adhesive capacity in the urinary tract. To confirm this preventive potential on urinary tract infections in children, a second study was carried out two years later over a period of one year. In this study, 40 children (39 girls and 1 boy) received cranberry juice daily, with and without PACs [161]. Children who participated in this study were those with at least two urinary tract infections, while those with anatomical diseases were excluded. One year of treatment with PACs at high concentrations led, as a major result, to a reduction in the risk of urinary tract infections (65%) [161].

On the other hand, with the aim of improving the durability of dentin bonds, Yi et al. [162] investigated the antibacterial effect of baicalein in association with ethanol-wet bonding. To this end, this flavone was dissolved in increasing concentrations of ethanol (0, 0.01%, 0.05%, and 0.1%) to treat 63 healthy human molars. The activity of these solutions was studied against *S. mutans*, since this bacterium is the main cariogenic agent [163]. Results revealed a dose-dependent antibiofilm effect, as well as a decrease in the total biomass area of the strain tested. Similarly, the effect of EGCG on two microorganisms responsible for dental caries in children has recently been studied [164]. In this study, 47 children susceptible to developing dental caries were selected to rinse their teeth with EGCG for one minute. From the enumeration of colony-forming units, a significant decrease in the concentrations of *Lactobacilli* and mutant *Streptococci* was observed in children [164].

## 6. Techno-Economic Challenges and Future Perspectives

Research related to the anti-QS drugs derived from natural sources may lead to the development of novel antibiotics with QS effects. However, although such advancement will lead to remarkable economic and health benefits, it will also face great technological challenges because the emergence of resistant bacteria is spreading globally, thus endangering the efficacy of antibiotics. In this context, different future perspectives should be attempted through further biological and pharmacological properties, clinical applications, and toxicological validations of new naturally derived drugs. Such development requires a mechanistic understanding of how the QS system functions and understanding of its molecular pathways. In this respect, although the QS system has been widely investigated, its implications in different bacterial phenotypes, particularly in the development of resistance against antibiotics, is not completely clear, because involved mechanisms are still not well developed.

## 7. Conclusions

Secondary metabolites from medicinal plants exhibit important antibacterial effects against several bacterial strains. Data collected through this review show that terpenoids, flavonoids, and phenolic acids exhibit numerous mechanisms, such as alteration of cell morphology, disturbance of cell membrane, decreasing membrane permeability, and inhibition of QS. These molecules could be considered as alternative drug candidates to conventional antibiotics. Indeed, these drugs exhibit quorum-quenching effects with several mechanisms, including inhibition of the production, the action, and the transport of QS mediators. On the other hand, these compounds showed potential results in clinical trials suggesting their possible use in therapeutic treatment of infectious diseases against resistant strains. However, clinical investigations require further studies to validate their use in humans. Antibiotic resistance has rapidly evolved in the last few decades to become one of the greatest public health threats of the 21st century. Indeed, infections that are untreatable due to multidrug resistance of the infected organism have become more common in clinical settings. A complete understanding of the mechanisms by which bacteria become resistant to antibiotics is of paramount importance to design novel strategies to counter the resistance threat. Therefore, efforts to develop antibiotics and study mechanisms of resistance should be continuous, resilient, and steady.

## Figures and Tables

**Table 1:** Antibacterial mechanisms of action of Terpenoids.

Molecules	Bacterial Species	Experimental Approaches	Key Results	References
Linalool	*Acinetobacter* *baumannii*	Evaluation of biofilm formation Anti-QS activity assay Quantification of biofilm biomass–CV staining	Inhibited biofilm formation Modified bacterial adhesion to surfaces Interfered with the QS system	[40]
Linalool	*Pseudomonas* *aeruginosa*	Determination of cell membrane permeability, membrane potential, and respiratory chain dehydrogenase	Damaged the respiratory chain Destroyed the integrity of bacterial membrane Disturbed normal cell morphology	[41]
Linalool nanoemulsions	*Salmonella* *typhimurium*	Biofilm inhibition studies Cell membrane integrity	Destroyed the integrity of bacterial membrane Induced high antibiofilm activity	[42]
(+)-Limonene	*Escherichia coli* BJ4	Cell permeabilization test	Induced permeabilization of bacterial membrane	[43]
(+)-Limonene	*Escherichia coli* lptD4213	Cell permeabilization test	Induced sublethal damage in the cytoplasmic membrane (at pH 4.0)	[43]
Limonene	*Listeria* *monocytogenes*	SEM analysis Conductivity measurement Determination of the effect of limonene on the respiratory chain complex I–V	Increased cell membrane permeability Destroyed the cell integrity and bacterial wall structure Affected respiration and energy metabolism	[44]
Limonene	*Streptococcus* *pyogenes* (SF370)	Analysis of antibiofilm potential, SEM, and cell viability assay	Reduced biofilm formation in a dose-dependent manner	[45]
Limonene	*Streptococcus* *mutans*	Analysis of antibiofilm potential, SEM, and cell viability assay	Inhibited acid production and downregulated the *vicR* gene Targeted the surface-associated proteins, thus reducing surface-mediated virulence factors	[45]
Phytol	*Pseudomonas* *aeruginosa*	Membrane depolarization assay DNA damage detection NAD^+^ cycling assay ROS measurement	Increased intracellular ROS level Increased transient depletion of NADH Induced DNA damage by oxidative stress Induced membrane depolarization Triggered inhibition of cell division	[46]
l-carvone	*Hafnia alvei*	In silico analysis RT-qPCR studies QS interference approach	Inhibited QS activity by reducing AHL production (0.5 μL/mL), biofilm formation (52.41%), swarming motility (74.94%), and swinging motility (61.49%)	[47]
Carvacrol	*Escherichia coli* and *Staphylococcus aureus*	Antibiofilm activity SEM analysis	Reduced biofilm formation	[48]
Carvacrol	*Salmonella enterica* serotype Typhimurium	MTT assay Crystal violet assay SEM analysis	Shriveled and retracted appearance at 4 × MIC Reduced metabolic activity (0.089 OD_550_) Reduced biofilm biomass (1.719 OD_550_)	[49]
Carvacrol and thymol	*Escherichia coli*	Fluorescent dyes Flow cytometry analysis	Disturbed cytoplasmic membrane	[50]
Carvacrol	*Listeria* *monocytogenes*	TEM analysis Flow cytometric analysis	Disrupted the structure of bacterial cells Induced degenerative changes in the cytoplasmic membrane and cell wall Modified respiratory activity Increased membrane permeability and depolarization	[51]
Geraniol	*Staphylococcus* *aureus*	Antibiofilm activity Biofilm biomass quantification	Reduced biofilm biomass. Reduced cell viability	[52]
Myrtenol	Methicillin-resistant *Staphylococcus aureus*	Extraction of staphyloxanthin Autolysis assay Ring biofilm inhibition assay	Inhibited production of staphyloxanthin Inhibited the synthesis of major virulence factors Inhibited biofilm formation	[53]
Myrtenol	*Staphylococcus* *aureus*	Antibiofilm effect	Inhibited biofilm formation	[54]
1,8-Cineole	*Salmonella* sp. D194-2	TEM analysis Proteomics analysis	Damaged the structure of cell walls and membranes Downregulated the carbohydrate metabolism and membrane protein-related genes	[55]
β-Caryophyllene	*Streptococcus* *mutans*	Confocal laser scanning microscope Real-time RT-PCR	Inhibited biofilm formation Reduced the expression of *gtf* genes	[56]
β-caryophyllene	*Bacillus cereus*	Measurement of UV-absorbing materials Zeta-potential measurement	Altered the membrane permeability and integrity	[57]

**Table 2:** Anti-quorum-sensing effects of terpenoids.

Compounds	Bacteria	Effects	References
Carvacrol	*Chromobacterium violaceum*	Inhibition of biofilm formation at sublethal concentrations Reducing of *cviI* expression Decreasing violacein and chitinase activity	[106]
	*Pseudomonas aeruginosa*	Inhibition of biofilm formation Reducing pyocyanin and violacein production	[107]
	*Pseudomonas aeruginosa* ATCC 10154	Reducing production of AHLs Reducing the expression of *lasR* expression Reducing biofilm formation	[108]
Sesquiterpene lactone	*Pseudomonas aeruginosa* ATCC 27853	Inhibition of QS phenotypes, such as biofilm formation, elastase activity, and AHLs	[109]
	*Chromobacterium violaceum*	Decreasing the affinity of CviR protein to its receptor LuxR	[110]
	*Chromobacterium violaceum* ATCC 12472	Inhibition of QS mediators	[111]
Eugenol	*Pseudomonas aeruginosa*, *Proteus mirabilis*, and *Serratia marcescens*	Reducing AHL and violacein formation	[112]
	*Escherichia coli* *Pseudomonas aeruginosa*	Decreasing violacein, elastase, pyocyanin, and biofilm formation Inhibition of *las* and *pqs* QS systems	[113]
	Methicillin-resistant *Staphylococcus aureus*	Reducing production of elastase, protease, chitinase, and pyocyanin	[114]
	*Pseudomonas aeruginosa*	Inhibition of biofilm formation	[115]
	*Pseudomonas aeruginosa*	Decreasing *rhlA*, *lasI*, and *rhlI* expression Inhibition of biofilm formation	[116]
Phytol	*Pseudomonas aeruginosa* PAO1	Inhibition of biofilm formation and pyocyanin production Reducing bacterial flagella motility	[117]
	*Serratia marcescens*	Inhibition of protease and biofilm production	[118]
	*Serratia marcescens*	Inhibition of biofilm, lipase, and hemolysin formation Inhibition of bacterial motility Downregulation of *fimA*, *fimC*, *flhC*, *flhD*, *bsmB*, *pigP*, and *shlA* genes expression Decreasing production of lipase and protease	[119]
Linalool	*Acinetobacter baumannii*	Inhibition of biofilm formation	[40]
d-limonene	*Escherichia coli*	Inhibition of biofilm formation Suppression of curli production Decreasing swimming and swarming ability	[120]
(−)-α-Pinene	*Campylobacter jejuni*	Reducing the QS communication	[121]

**Table 3:** Anti-quorum-sensing effects of flavonoids.

Compounds	Organisms Tested	Key Findings	References
Epigallocatechin	*Burkholderia cepacia* and *Staphylococcus aureus*	Inhibited biofilm formation by interference with AHL production	[123]
	*Eikenella corrodens*	Inhibited QS mediated by auto-inducer 2 (AI-2) Inhibited biofilm formation	[124]
	*Listeria monocytogenes*	Inhibited biofilm formation	[125]
	*Campylobacter jejuni*	Disturbed QS functionin Reduced motility and biofilm formation Decreased AI-2 activity	[126]
	*Streptococcus mutans* (Sm) and probiotic *Lactobacillus casei* in Yakult (LcY)	Decreased biomass and acid production Inhibited biofilm formation Acid production	[127]
	*Salmonella typhimurium*	Reduced *sdiA* and *luxS* genes expression	[128]
Naringin	*Chromobacterium violaceum*	Inhibited biofilm formation Reduced swimming and swarming motility Inducted some gene transcription, such as *yenR*, *flhDC*, and *fliA*	[129]
	*Yersinia enterocolitica*	Inhibited biofilm formation Decreased the synthesis of AHL	[129]
	*Streptococcus mutans*	Suppressed biofilm maturation	[130]
Quercetin	*Escherichia coli* O157:H7 and *Vibrio harveyi*	Inhibited biofilm formation Blocked cell–cell signaling	[83]
	*Chromobacteriumviolaceum* CV026	Reduced violacein production, biofilm formation, EPS production, motility, and alginate production	[131]
	*Pseudomonas aeruginosa* PAO1	Inhibited biofilm formation Inhibited the twitching motility	[79]
	*Pseudomonas aeruginosa* strain PAO1	Inhibited biofilm formation Reduced virulence factors, including pyocyanin, protease, and elastase Reduced levels of *lasI*, *lasR*, *rhlI*, and *rhlR* genes expression	[132]
Quercetin 4’-*O*-β-d- glucopyranoside	*Chromobacteriumviolaceum* CV12472 and *Pseudomonas aeruginosa* PAO1	Inhibited violacein, elastase, pyocyanin, and biofilm formation	[133]
	*Pseudomonas aeruginosa*	Inhibited LasR expression	[134]
	*Chromobacterium violaceum* ATCC 12,472 and *Chromobacterium violaceum* CV026	Inhibited production of violacein pigment Inhibited the communication molecule, C6-AHL	[135]
	*Pseudomonas aeruginosa*	Decreased adhesion, biofilm formation, swarming motility, and expression of biofilm-associated genes Reduced pyocyanin production Inhibited the activity of protease Reducing QS via the *vfr*-mediated *lasIR* system	[136]
Taxifolin	*Pseudomonas aeruginosa* PAO1	Reduced production of pyocyanin and elastase Inhibited the QS-controlled genes expression	[137]
Kaempferol	*Staphylococcus aureus*	Inhibited biofilm formation Inhibition of adhesion-related gene expression	[138]
Morin	*Staphylococcus aureus*	Inhibited biofilm formation Reduced motility and spreading	[139]
Naringenin	*Pseudomonas aeruginosa*	Inhibited the QS-regulated gene expression	[140]

**Table 4:** Anti-QS effects of phenolic acids.

Compounds	Organisms Tested	Key Findings	References
Rosmarinic acid	*Pseudomonas aeruginosa* PAO1	Inhibited biofilm formation	[141]
	*Pseudomonas aeruginosa* PAO1	Inhibited QS regulator RhlR and *N*-butanoyl- homoserine lactone (C4-HSL) Induced a great increase in RhlR-mediated transcription than that of C4-HSL Induced QS-dependent gene expression Inhibited biofilm formation and virulence factor production (pyocyanin and elastase)	[142]
	*Aeromonas hydrophila*	Biofilm inhibitory concentration was 750 μg/mL Reduced production of QS-mediated hemolysin, lipase, and elastase Downregulated the virulence genes, such as *ahh1*, *aerA*, *lip*, and *ahyB*	[143]
	*Pseudomonas aeruginosa* PAO1	Induced the expression of 128 genes, including numerous virulence factor genes Induced seven sRNAs that were all encoded in regions close to QS-induced genes	[144]
Chlorogenic acid	*Pseudomonas aeruginosa*	Inhibited biofilm formation, swarming, and virulence factors Downregulation of QS-related gene expression Inhibition of QS receptors	[145]
	*Chromobacterium violaceum*	Inhibited biofilm formation, swarming motility, chitinolytic activity, and violacein production	[145]
Salicylic acid	*Agrobacterium tumefaciens*	Decreased biofilm and AHL production via the modulation of 103 genes’ expression	[146]
	*Pseudomonas aeruginosa*	Decreased swimming, twitching, and swarming motility	[147]
	*Pectobacterium carotovorum* and *Pseudomonas syringae* pv *syringae*	Inhibited biofilm formation, motility, and AHL production	[148]
	*Pectobacterium aroidearum* and *Pectobacterium carotovorum* ssp. *brasiliense*	Affected the QS machinery of the two species, consequently altering the expression of bacterial virulence factors Inhibited QS genes’ expression, such as *expI*, *expR*, *PC1_1442* (*luxR* transcriptional regulator), and *luxS* (a component of the AI-2 system) Reduced AHL levels	[149]
Cinnamic acid	*Pseudomonas aeruginosa* PAO1	Inhibited QS-dependent virulence factors and biofilm formation	[150]
	*Pectobacterium aroidearum* and *Pectobacterium carotovorum* ssp. *brasiliense*	Altered gene expression of virulence factors Inhibited genes expression of QS (*expI*, *expR*, *PC1_1442* (*luxR* transcriptional regulator), and *luxS*) Decreasing the expression of AHL signal	[149]
Two cinnamic acid derivatives: 4-dimethyl-aminocinnamic acid and 4-methoxycinnamic acid	*Chromobacterium violaceum* ATCC12472	Inhibited the synthesis of *N*-decanoyl-homoserine lactone Reduced production of virulence factors (violacein, hemolysin, and chitinase) Downregulated some QS-related metabolites (ethanolamine and l-methionine) Decreased QS-related genes expression (*cviI* and *cviR*) Inhibited biofilm formation	[151]
*p*-Coumaric acid	*Agrobacterium tumefaciens* NTL4, *Chromobacterium violaceum* 5999, and *Pseudomonas chlororaphis*	Inhibited QS responses	[152]
	*Chromobacterium violaceum* (CECT 494)	Inhibited the production of violacein	[153]
Caffeic acid	*Staphylococcus aureus*	Reduced bacterial adhesion Decreased the production of α-hemolysin	[154]
Ellagic acid	*Burkholderiacepacia*	Inhibited biofilm formation	[123]
Phenylacetic acid	*Pseudomonas aeruginosa*	Exhibited competitive action with AHLs signaling Decreased the production of pyocyanin, protease, and elastase	[155]

**Table 5:** Clinical trials of terpenoids as antibacterial drugs.

Molecules	Treatment	Experimental Approaches	Bacterial Strains	Key Results	References
Thymol + eugenol	One douche/day for one week	A multicenter, parallel group, randomized study 221 bacterial vaginosis cases	Vaginal strain	Reduced the severity of dyspareunia, vaginal dryness, erythema, and itching Reduced vaginal pH	[158]
Thymol + chlorhexidine	T_0_, before general anesthesia; T_1_, one month after treatment; T_2_, six months after treatment; T_3_, twelve months after treatment	90 patients randomly assigned into 3 groups Caries risk test Bacterial counts for each individual patient at four stages (T_0_, T_1_, T_2_, and T_3_)	Salivary *mutans streptococci* and *lactobacilli*	Decreased bacterial values compared to the control group No significant differences at T_0_ and T_3_	[159]
β-caryophyllene	126 mg/day for eight weeks	Randomized double-blind, placebo-controlled trial 33 patients received β-caryophyllene 33 patients received a placebo preparation	*Helicobacter* *pylori*	No significant change in the urea breath test Improvement of epigastralgia and nausea Decreased serum IL-1β levels	[157]
